# Isosorbide mononitrate promotes angiogenesis in embryonic development of zebrafish

**DOI:** 10.1590/1678-4685-GMB-2019-0233

**Published:** 2020-07-24

**Authors:** Hui Lv, Bo Liu, Yongwen Qin

**Affiliations:** 1 The Second Affiliated Hospital of ShanXi Medical University, Department of Cardiovascular Disease, Taiyuan, Shanxi, 030001, China.; 2 Xinhua Hospital Affiliated To Shanghai Jiaotong University School Of Medicine, Department of Cardiovascular Disease, Shanghai 200092, China.; 3 The Second Military Medical University, Department of Cardiovascular Disease, Shanghai 200433, China

**Keywords:** Isosorbide mononitrate, angiogenesis, coronary heart disease, zebrafish, miRNA

## Abstract

Coronary heart disease (CHD) is a leading cause of death worldwide, and angiogenesis plays important roles in CHD. Thus, in the present study, the angiogenic efficacy of four common cardiovascular medicines (aspirin, pravastatin, metoprolol and isosorbide mononitrate (ISMN)) was determined by the number and length of zebrafish intersegmental vessels (ISVs) after immersing zebrafish embryos in different medicines. Results showed that ISMN significantly increased the length and number of ISVs. ISMN is a long-acting nitrate ester drug. It has been used as a vasodilator to dilate arteries and veins to reduce the cardiac preload and postload. However, the effect of ISMN on angiogenesis remains unclear. Thus, by in vitro experiments, the angiogenic mechanism of ISMN was evaluated through detecting the viability and proliferation of human umbilical vein endothelial cells (HUVECs) and the expression of angiogenesis-related genes and miRNAs. Results indicated that ISMN could increase the viability and proliferation of HUVECs by decreasing apoptosis, and elevated the expressions of *vedf*, *kdrl*, *pdgfr* in zebrafish embryos. Furthermore, the expressions of miR-126, miR-130a and miR-210 were also regulated in ISMN-treated HUVECs. In conclusion, ISMN could promote angiogenesis in zebrafish embryos and HUVECs, implying ISMN may be a potential therapeutic in treating angiogenesis-related diseases.

## Introduction

Coronary heart disease (CHD) is still the major cause of death in the world even if the past 20 years witnessed a dramatic decline (Colquhoun *et al.*, 2000; Capewell and O’Flaherty, 2008; Jones and Greene, 2013). In recent decades, research found that angiogenesis plays central roles in the pathological process of CHD (Zhang *et al.*, 2018). Therapeutic angiogenesis is an alternative approach to augment the innate myocardial angiogenesis and formation of collateral circulations following ischemia for the no-option patients (Grass *et al.*, 2006). Angiogenesis was modulated by a subset of signaling pathways including vascular endothelial growth factor (Vegf)-Vegfr, Tie-angiopoietin, transforming growth factor (TGF)-beta, platelet-derived growth factor (Pdgf), and integrins (Warren and Iruela, 2010). Meanwhile, in recent years, miRNAs have been reported to regulate various stages of angiogenesis. MiRNAs are 21-23 nucleotides long, single stranded noncoding RNA molecules. MiRNA’s capacity to target genes within a signaling pathway makes them promising target for anti-angiogenesis drugs (Tiwari *et al.*, 2018). It is demonstrated that a few specific miRNAs, such as miR-126, miR-210, 221 and 222, could regulate angiogenesis process (Poliseno *et al.*, 2006; Chen and Gorski, 2008; Urbich *et al.*, 2008).

Previously, studies demonstrated that statins, a kind of cardioprotective drug, could suppress the growth of cancer cells through its antiangiogenic activities (Wang *et al.*, 2010), and induce angiogenesis in stroke and ischemic heart disease (Elewa *et al.*, 2012). Moreover, other types of cardiovascular medicines, such as antiplatelet drug, β_1_ receptor blocker and nitrates, also have potentials to regulate angiogenesis (Ulu *et al.*, 2009; Su *et al.*, 2014; Rammos, 2015). But, the efficacy and mechanism of these cardioprotective drugs on the therapeutic angiogenesis remain unclear. Thus, in the present study, we selected pravastatin (a statin), aspirin (an antiplatelet drug), metoprolol (a β_1_ receptor blocker) and ISMN (a nitrate) to test their angiogenic abilities.

To investigate the roles of cardiovascular medicines in angiogenesis and identify the underlying mechanisms, in this study, *Tg(flk1:eGFP)* zebrafish embryo angiogenesis model and human umbilical vein endothelial cells (HUVECs) were employed. The angiogenic efficacy of different types of cardioprotective drugs was determined by the number and length of zebrafish intersegmental vessels (ISVs). Here, we preliminarily noticed that ISMN significantly enhanced the ISV growth. As ISMN belongs to nitrates, we further investigated whether nitrates can promote angiogenesis. Hence, different kind of nitrates, including nitroglycerin (NTG), isosorbide dinitrate (ISDN) and ISMN were further used to test their roles in angiogenesis. Among these nitrates, ISMN increased the length and number of ISV significantly, thus, the angiogenic mechanism of ISMN was further evaluated by detecting apoptosis in HUVECs and the expressions of *vedf*, *kdrl*, *flt-4*, *fli1a* and *pdgfr* in zebrafish embryos. Thereafter, the expressions of miR-126, miR-130a, miR-210, miR-221 and miR-222 were also measured.

## Material and Methods

### Zebrafish embryo collection and drug administration

Tg(flk:eGFP) zebrafish embryos, in which vascular endothelial cells are fluorescently stained with the enhanced green fluorescent protein (eGFP), were generated by natural pairwise mating and were maintained at 28.5 °C in embryo water as described by Westerfield (Cross *et al.*, 2003). Different types of drugs were dissolved in 0.5% Dimethyl sulfoxide (DMSO) to form different concentration of solutions. The concentration of drugs was based on the blood drug level tested in human being. Zebrafish embryos were arrayed in 24-well plate and incubated with 2 mL solution containing various concentration per well at 28.5 °C for 24 h. Embryos incubated in DMSO (0.5%) were served as control. Each trial was performed with three times of repetition at least, with 25 embryos each group. The length of ISVs was measured by the software provided by LEICA company which can calculate point to point distances.

### Cell line and cell culture

Human Umbilical Vein Cells (HUVEC) (ATCC, VA, USA) were maintained in DMEM medium (Invitrogen, CA, USA) supplemented with 2% fetal bovine serum, 100 U/mL penicillin and 100 μg/mL streptomycin at 37 °C with 5% CO_2_ (Lam *et al.*, 2008).

### RNA extraction

Total RNA from zebrafish embryos at 0, 6, 12, 24 hpf was isolated using Trizol-Reagent according to the manufacture’s protocol. Ten zebrafish embryos or about 1×10^5^ cells were harvested from each group. The quantity and quality of RNA were estimated by spectrophotometer (Qiagen, Germany). The ratio of OD_260_/OD_280_ should be above 1.8.

### Quantitative real-time PCR (qRT-PCR) analysis

The total RNA extracted from zebrafish embryos or cells were reverse transcribed to cDNA by using PrimeScript RT reagent Kits (TaKaRa, Dalian, China) with special stem-loop primer for miRNA and oligo-dT or random primer for mRNA. qRT-PCR was performed on a Rotor-Gene instrument (Qiagen, Germany) using SYBR Green. The primers used in the amplification were listed in the Table S1 and Table S2. The housekeeping genes *U6* and *gapdh* were applied as internal standards for miRNAs and mRNA, respectively. The cycling program was set as follows: 94 °C, 15 s, 58 °C, 15 s, 72 °C, 20 s, 40 cycles. Relative abundance was calculated by the delta-delta Ct method.

### MTT assay for cell viability

The assessment of cell vitality was performed by MTT assay. Briefly, HUVECs were seeded into 96-well cell culture plates at an initial density of 1×10^4^ cells/well. Following a 24 h treatment of 4 ng/mL, 20 ng/mL, 0.1 μg/mL, 0.5 μg/mL and 5 μg/mL ISMN, 20 μL of a 5 mg/mL solution in PBS of the MTT substrate (Sigma-Aldrich, MO, USA) was added and incubated for up to 4 h. The resulting blue-brown formazan precipitate formed was solubilized by DMSO. A curve of cell vitality was constructed by measuring cell growth with a microplate reader at 490 nm.

### Cell proliferation

The surface of each well of the 48-well cell culture dish was coated with 200 μL Matrigel Matrix (BD Biosciences). Then, the culture dish was incubated for 2 h at 37 °C to solidify the Matrigel. HUVECs were spread at 2×10^4^ cells/well. They were then treated with ISMN at 0.05 μg/mL, 0.5 μg/mL and 5 μg/mL respectively for 6 h, and the images of cells were captured by a CCD camera (AxioCam HC, Carl Zeiss, Thornwood, NY). Eight fields were randomly selected and the cell number was counted by Image J software (National Institutes of Health; http://rsbweb.nih.gov/ij/).

### Flow cytometry assay for cell apoptosis

After treated with 5 μg/mL ISMN for 24 h, adherent HUVECs were released by trypsinization. The cell apoptosis was investigated by Annexin V: FITC kit (Roche, Basel, Switzerland) based on the manufacturer’s specifications. The samples were analyzed by a Becton Dickinson FACS Aria cell sorter.

### Statistical analysis

The data was presented as mean ± SD. Data was analyzed using SPSS software 16.0 (SPSS Inc., Chicago, IL). Statistical significance was assessed by one-way ANOVA. *P* < 0.05 were considered as statistically significant.

## Results

### ISMN accelerated blood vessel formation

**Figure 1:**
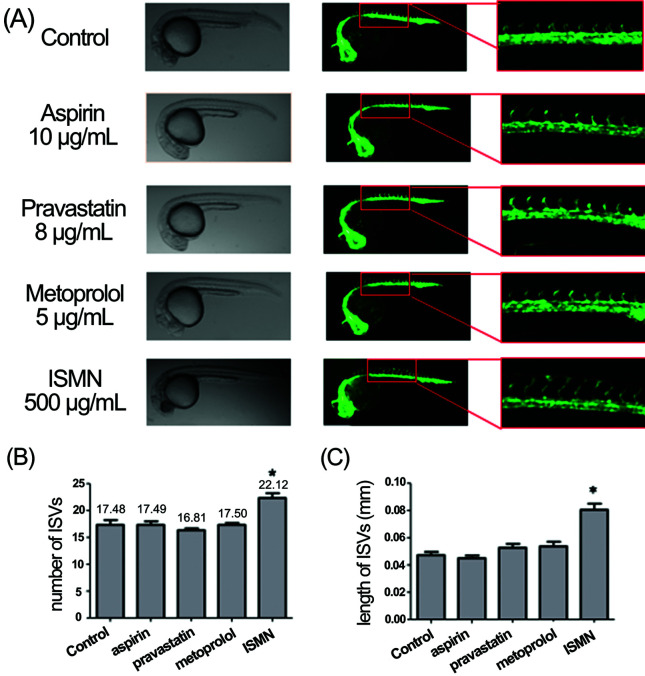
Screening results of cardiovascular drugs with angiogenic ability using *Tg(flk:EGFP)* zebrafish. (A) Fluorescence images of 24 hpf zebrafish embryos treated with cardiovascular drugs for 24 h. (B) The number of ISVs in zebrafish embryos. (C) The length of ISVs in zebrafish embryos. Data were expressed as mean ± SD (n = 3). * *p* < 0.05.

To figure out the effects of cardiovascular medicines on angiogenesis, we first examined the vessel formation of zebrafish embryos. *Tg(flk1:eGFP)* zebrafish embryos (n = 25 each group) were treated with 4 types of drugs, including aspirin (antiplatelet drug), pravastatin (statins), metoprolol (β_1_ receptor blocker) and ISMN (nitrates). There was no obvious difference in the development of dorsal aorta among the 4 treatment groups at 12 hours post fertilization (hpf) and 24 hpf (data not shown). However, the number of intersegmental vessels (ISVs) was significantly increased in the ISMN group compared with that in the other 3 groups (*p* < 0.05) (Figures 1A and 1B). Furthermore, as shown in Figure 1C, the length of ISVs in ISMN-treated embryos was significantly elevated compared with that in the control group (*p* < 0.05), whereas, other drugs showed no significant effect on the ISVs growth.

### ISMN promoted angiogenesis

**Figure 2:**
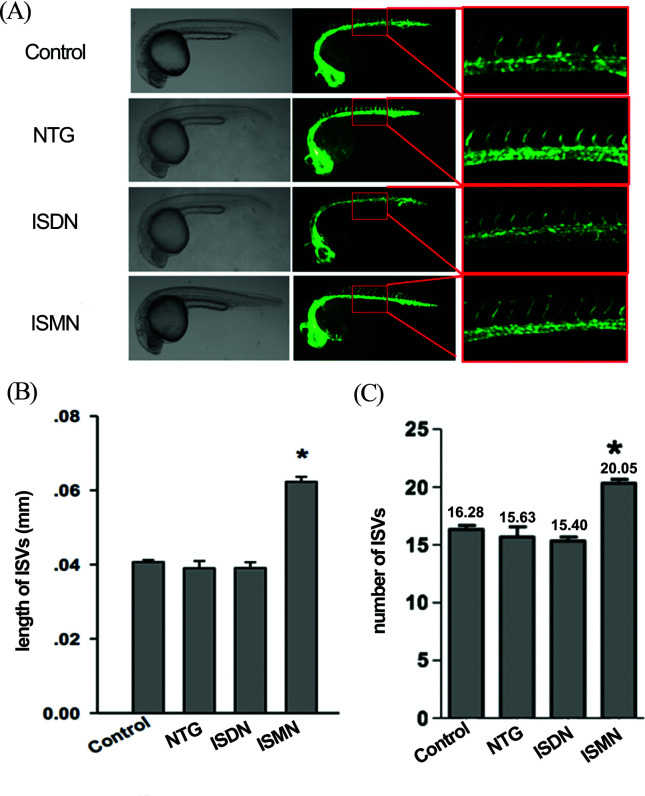
Effects of nitrates on angiogenesis of ISVs. (A) Fluorescence images of zebrafish embryos at 24 hpf treated with DMSO (control) and different nitrates. (B) The number of ISVs in zebrafish embryos treated with nitrates. (C) The length of ISVs in zebrafish embryos treated with nitrates. Data were expressed as mean ± SD (n = 3). * *p* < 0.05.

According to the above findings, as a nitrate, ISMN could promote ISVs growth. Moreover, in a previous study, researchers found that nitric oxide donors could be successfully used for the treatment of developed angiogenesis-inhibitor-induced hypertension (Kruzliak *et al.*, 2013). Thus, we speculate that some nitrates may also play a role in angiogenesis. Therefore, we further explored the impacts of various common nitrates cardioprotective drugs on the growth of ISVs. Nitroglycerin (NTG), isosorbide dinitrate (ISDN) and ISMN were used to treat zebrafish embryos thrice, twice and once a day independently according to their pharmacokinetics in human. Interestingly, compared with the control embryos, the lengths and numbers of ISVs were only significantly increased in ISMN-treated group at 24 hpf (*p < 0.05*) (Figure 2).

### ISMN treatment up-regulated expressions of *vegf-a*, *kdrl*, and *pdgfr* in zebrafish embryos

**Figure 3:**
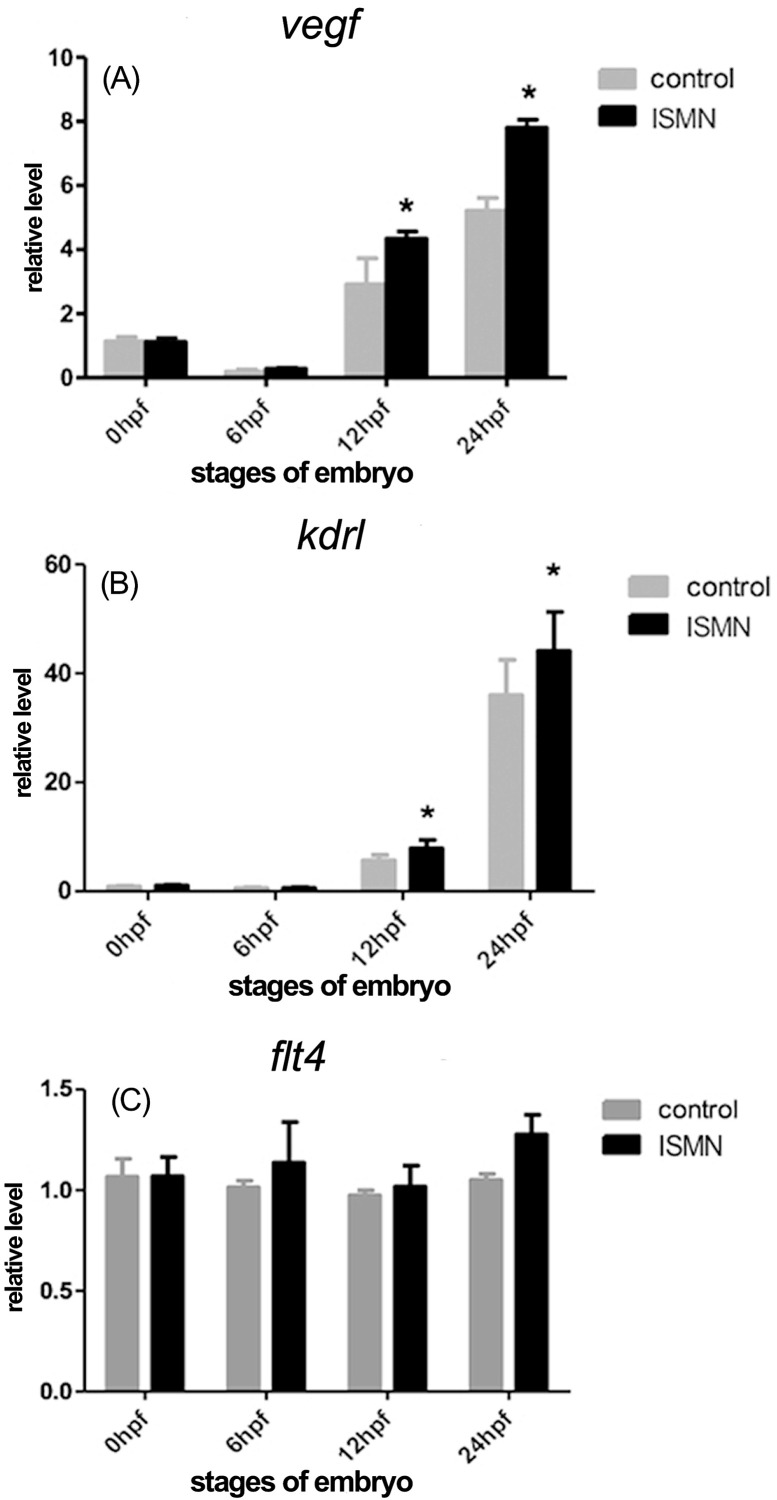
Analysis of target genes involved in VEGF signaling pathways in ISMN-treated zebrafish embryos. (A) Relative expression of *vegf-a*. (B) Relative expression of *vegfr-2* (*kdrl*). (C) Relative expression of *vegfr-3* (*flt4*). Data were expressed as mean ± SD (n = 3). * *p* < 0.05.

**Figure 4:**
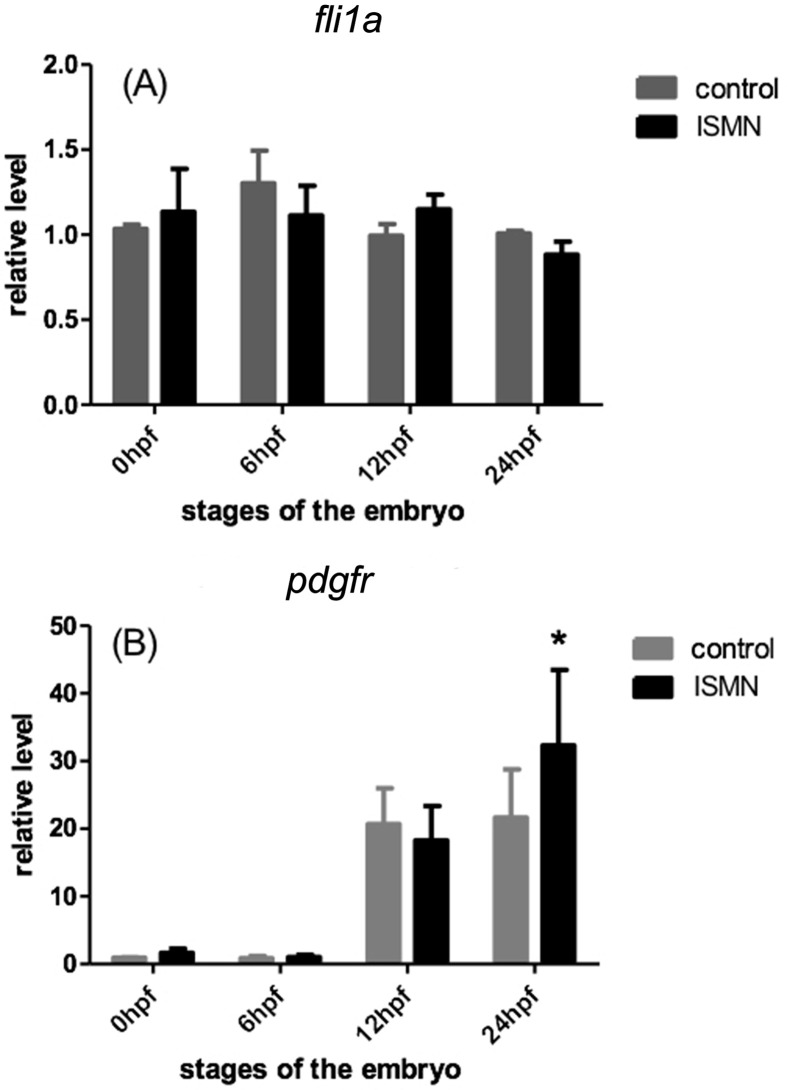
Expressions of *fli1a* and *pdgfr* in zebrafish embryos treated with ISMN for 24 h. (A) *fli1a* expression. (B) *pdgfr* expression. Data were expressed as mean ± SD (n = 3). * *p* < 0.05.

In order to investigate the molecular mechanisms of ISMN-induced angiogenesis, expression levels of selected genes involved in angiogenesis process were detected at a series of incubation time using qRT-PCR. Vegf-a, a major regulator for angiogenesis, could bind and activate Vegfr2 (Kdrl) in zebrafish (Schuermann *et al.*, 2014). Figure 3 shows that ISMN could significantly increase mRNA levels of *vegf-a* and *kdrl* at 12 hpf and 24 hpf (*p* < 0.05). No significant changes were observed in *vegfr3* (*flt4*) mRNA expression. Fli1a, one factor of ETS domain gene, also links with angiogenesis (Brown *et al.*, 2000). Figure 4A indicates that ISMN could not alter the *fli1a* expression level significantly. Moreover, *pdgfr*, which can affect vascular development, was up-regulated in ISMN treated embryos at 24 hpf (*p* < 0.05) (Figure 4B). Hence, these results suggest that the up-regulation of *vegf-a*, *kdrl*, and *pdgfr* expressions by ISMN may contribute to the pro-angiogenesis in zebrafish embryos.

### ISMN increased HUVECS viability and proliferation and decreased HUVECS apoptosis

**Figure 5:**
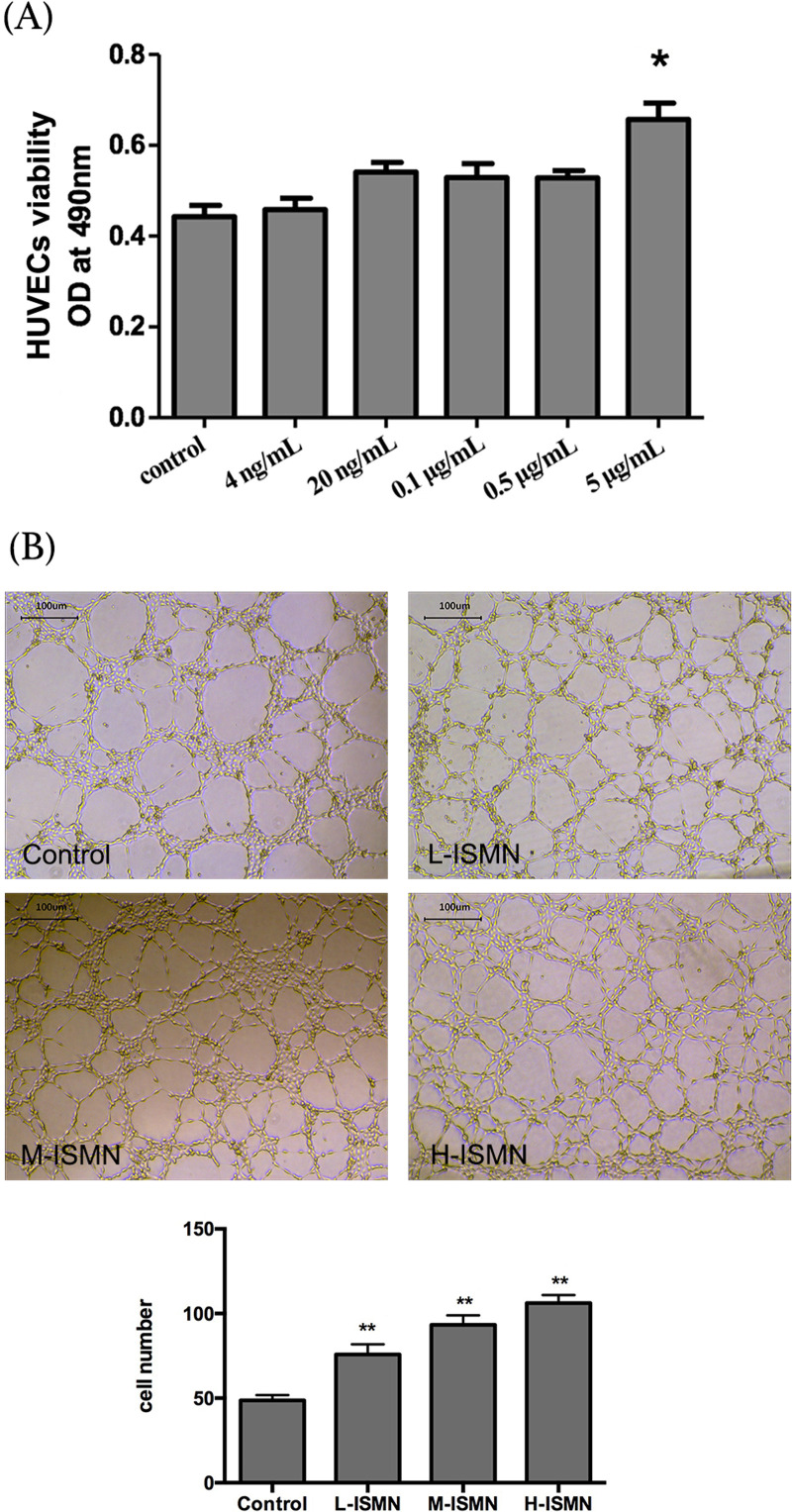
Effects of ISMN on HUVECs viability and proliferation. (A) HUVECs viability determined by MTT. (B) HUVECs proliferation. L-ISMN, 0.05 μg/mL; M-ISMN, 0.5 μg/mL; H-ISMN, 5 μg/mL. DMEM treatment was used as control. Data were expressed as mean ± SD (n = 3). * *p* < 0.05.

**Figure 6:**
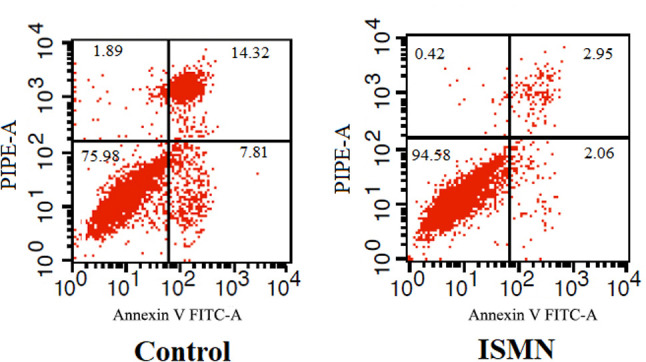
Effects of ISMN on HUVEC apoptosis by Annexin V-FITC/PI flow cytometry. Q1 (annexin V-/PI+) - cells with features of necrosis; Q2 (annexin V+/PI+) - cells with features of late apoptosis; Q3 (annexin V-/PI-) - viable cells; Q4 (annexin V+/PI-) - cells with features of early apoptosis.

The effects of ISMN at 4 ng/mL, 20 ng/mL, 0.1 μg/mL, 0.5 μg/mL, and 5 μg/mL on cell viability of HUVEC were assessed by MTT assay. As shown in Figure 5A, we observed that ISMN increased cell viability in a dose-dependent manner, and 5 μg /mL ISMN significantly elevated HUVECs viability (*p* < 0.05). Then, the proliferation of HUVECs was also investigated. Based on Figure 5B, after treating HUVECs with ISMN at 0.05 μg/mL, 0.5 μg/mL and 5 μg/mL, the cell numbers were significantly increased (*p* < 0.01) compared with the control group. To determine if the increased cell viability and proliferation were induced by ISMN was due to less apoptosis, Annexin-V staining was utilized (Figure 6). The average number of non-viable cells and AnnexinV^+^/PI^+^ cells in ISMN-treated HUVECs was decreased compared to the control ones (24.02% *vs* 5.42%; 14.32% *vs* 2.95%), indicating that ISMN could decrease apoptosis in HUVECs.

### Expression of miRNAs involved in angiogenesis in HUVECS induced by ISMN

**Figure 7:**
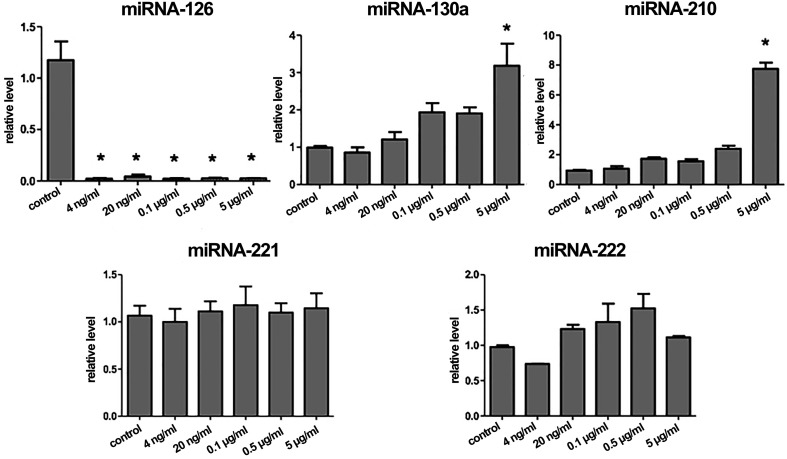
Effects of different levels of ISMN on miRNAs expressions in HUVECs after 24 h treatment. Data were expressed as mean ± SD (n = 3). * *p* < 0.05.

ISMN at different concentrations was used to treat HUVECs to analyze the expression of miRNAs involved in angiogenesis. As shown in Figure 7, the expression of miR-126 was significantly down-regulated by ISMN at all the tested concentrations (*p* < 0.05). Besides, 5 μg/mL ISMN could significantly enhanced the expressions of miR-130a and miR-210 (*p* < 0.05). Nevertheless, no significant changes in the expressions of miRNA-221 and miRNA-222 were observed (Figure 7). Altogether, these results suggested that the increased expressions of miR-130a and -210, and the decreased expression of miR-126 induced by ISMN promoted angiogenesis of HUVECs.

## Discussion

Angiogenesis therapy was once considered as an alternative to traditional revascularization in no-option patients, but recently, it has opened unprecedented opportunities for CHD treatment (Emanueli and Madeddu, 2006). The effects of pro-angiogenic therapy against ischemic diseases have been validated in various experiments (Cao, 2010). Beside, some cardiovascular medicines also show potentials to affect angiogenesis (Ulu *et al.*, 2009; Su *et al.*, 2014). However, the efficacy and mechanisms of these medicines in angiogenesis are still unclear.

In the current study, we investigated the angiogenic effects of different cardiovascular drugs, including aspirin, pravastatin, metoprolol and ISMN. ISMN, one of the most frequently used compounds for CHD treatment, is an organic nitrate vasodilator that can reduce myocardial oxygen demand and increase oxygen supply by vasodilating capacitance veins and coronary arteries (Stockis *et al.*, 2002). Here, we found that only ISMN could increase the number and the length of ISVs in zebrafish embryos, indicating the potential angiogenic ability of ISMN. Then, the above findings promoted us to explore whether other nitrates also have angiogenic abilities. It is reported that 24 hpf is an important time for ISVs formation (Ellertsdottir *et al.*, 2010). By using the clinically most relevant organic nitrates (NTG, ISDN and ISMN), we noticed that only ISMN showed an angiogenic effect at 24 hpf in zebrafish embroys, which may due to the different chemical structures of these nitrates (Csont and Ferdinandy, 2005).

Organic nitrates can exert their biological effects via the release of nitric oxide (NO) (Cao, 2010). NO can induce angiogenesis through regulating VEGF (Salvolini *et al.*, 2010). Vegf and Vegfrs are major contributors to zebrafish vascular development. In the Vegf family, Vegf and Vegfr-2 (Kdrl) play important roles in the angiogenesis and formation of collateral vessels (Yla-Herttuala *et al.*, 2007; Liu and Patient, 2008). In the present study, ISMN significantly increased the mRNA level of *vegf* and *vegfr2* (*kdrl*), which implies that the angiogenic effects of ISMN can be modulated by VEGF expression. Besides, Vegfr-3 (flt4) is mainly expressed in lymphatic endothelial cells (EC) (Lymboussaki *et al.*, 1998), but the *flt4* expression was not altered significantly by ISMN. Moreover, ISMN treatment also significantly up-regulated the expression of *pdgfr*, which is required for zebrafish angiogenesis process (Wiens *et al.*, 2010). Although fli1a is also essential for angiogenesis (Liu and Patient, 2008), ISMN did not change *fli1a* transcription level significantly. Therefore, the up-regulation of *vegf-a*, *kdrl*, and *pdgfr* expressions by ISMN may contribute to the pro-angiogenesis in zebrafish embryos.

Furthermore, by taking advantage of HUVECs, we further examined the mechanisms of ISMN on angiogenesis. The *in vitro* data indicates that ISMN increased HUVECs viability in a dose-dependent manner. Meanwhile, ISMN also enhanced the proliferation and decreased the apoptosis of HUVECs. Altogether, the angiogenic effect of ISMN may be achieved through the regulation of endothelial cell viability.

MicroRNAs are a class of conserved non-coding small RNAs, which can cause post-transcriptional inhibition of gene expression by targeting the 3’ UTRs of mRNAs (Bushati and Cohen, 2007). Much evidence indicates that miRNAs are key regulators in angiogenesis and endothelial function (Wang and Olson, 2009). MiR-126 is the only miRNA of EC-specific expression and miR-126 level is decreased when VEGF signaling pathway is activated (Zhu *et al.*, 2011). In the current study, we found that miR-126 expression was significantly down-regulated in HUVECs exposed to ISMN. Moreover, our data also showed that ISMN treatment significantly up-regulated the expression of miR-130a, which can down-regulate the anti-angiogenic protein and promote angiogenesis (Chen and Gorski, 2008). Additionally, miR-210, which can enhance the formation of capillary-like structures (Tiwari *et al.*, 2018), was shown to up-regulate in the present experiment. Among the miRNAs with higher expression in HUVECs, miR-221 and miR-222 were reported to resist angiogenesis (Poliseno *et al.*, 2006), but in our study, miR-221 and -222 had no obvious responses to ISMN treatment. Taken together, the increased expressions of miR-130a and -210, and the decreased expression of miR-126 induced by ISMN may play a role in angiogenesis.

In conclusion, our study revealed that ISMN could promote angiogenesis in embryonic development of zebrafish via regulating *vegf-a*, *kdrl*, and *pdgfr*. Moreover, by using HUVECs, we found that the decreased apoptosis, down-regulated miR-126 level and up-regulated levels of miR-210 as well as miR-130a may also contributed to angiogenesis. These findings deepen our understanding of the angiogenic ability of cardiovascular drugs in treating CHD. However, limitations exist in the present work. It remains to know whether inhibition and overexpression of the angiogenic genes could influence angiogenesis. Moreover, clinical trials should also be designed for therapeutic evaluation of the pro-angiogenic therapy with ISMN. Thus, future studies should be employed to further clarify the specific angiogenic mechanisms mediated by ISMN.

## Supplementary Material

The following online material is available for this article:

Table S1 Primers for genes involved in angiogenesis in zebrafish.

Table S2 Primers for miRNAs involved in angiogenesis in HUVECs.
