# Supplementary material for: Isosorbide mononitrate promotes angiogenesis in embryonic development of zebrafish
Source: Genet Mol Biol. 2020 Jul 24;43(3):20190233. doi: 10.1590/1678-4685-GMB-2019-0233 (PMC7380327; doi:10.1590/1678-4685-GMB-2019-0233)
Supplement: Supplementary file 1 [file 1415-4757-GMB-43-3-e20190233-suppl1.pdf]

Genetics and Molecular Biology
https://doi.org/10.1590/1678-4685-GMB-2019-0233

Supplementary Material to "Isosorbide mononitrate promotes angiogenesis in embryonic development of zebrafish"

Table S1 - Primers for genes involved in angiogenesis in zebrafish

Primers	Sequences (5'-3')
vegf R	CGAAACGTCACTATGGAGGTG
vegf F	GCAAGGCTCACAGTGGTTTT
flt4 R	ATGCCGGGGTATGGAGAC
flt4 F	CAGGCTTTAGTGAGGAGAGAGG
kdrl F	CTGGTGGAGAGGCTAGGAGA
kdrl R	TGATCGGGATGTAGTGCTTTC
fli1a R	CAGCATCATAAGACGCATCG
fli1a F	CCATCTCACACGCTGACCAGT
pdgfr R	TTCCCCTCGAGAGACAGGA
pdgfr F	GGCACACAGAAAAGGGAAAG
gapdh R	CATGTAATCAAGGTCAATGAATGG
gapdh F	CAGGCATAATGGTTAAAGTTGGTA
