# Supplementary material for: Isosorbide mononitrate promotes angiogenesis in embryonic development of zebrafish
Source: Genet Mol Biol. 2020 Jul 24;43(3):20190233. doi: 10.1590/1678-4685-GMB-2019-0233 (PMC7380327; doi:10.1590/1678-4685-GMB-2019-0233)
Supplement: Supplementary file 2 [file 1415-4757-GMB-43-3-e20190233-suppl2.pdf]

Genetics and Molecular Biology
https://doi.org/10.1590/1678-4685-GMB-2019-0233

Supplementary Material to “Isosorbide mononitrate promotes angiogenesis in embryonic development of zebrafish”

Table S2 - Primers for miRNAs involved in angiogenesis in HUVECs

Primers	Sequences（5’ -3’）
miR-210F	ACACTCCAGCTGGGCTGTGCGTGTGACAGC
miT-210R	CTCAACTGGTGTCGTGGAGTCGGCAATTCAGTTGAGTCAGCCGC
miR-222F	ACACTCCAGCTGGGAGCTACATCTGGCTGGCTACTG
miR-222R	CTCAACTGGTGTCGTGGAGTCGGCAATTCAGTTGAGGAGACCCA
miR-130aF	ACACTCCAGCTGGGCAGTGCAATGTTAAAA
miR-130aR	CTCAACTGGTGTCGTGGAGTCGGCAATTCAGTTGAGATGCCCTT
miR-221F	ACACTCCAGCTGGGAGGGAGCTACATTGTCTGCT
miR-221R	CTCAACTGGTGTCGTGGAGTCGGCAATTCAGTTGAGAAACCCAG
miR-126F	ACACTCCAGCTGGGAGGCGCTCGTACCGTGAGTAA
miR-126R	CTCAACTGGTGTCGTGGAGTCGGCAATTCAGTTGAGAGGACATT
U6 F	GTGCTCGCTTCGGCAGCACAT
U6 R	ATGGAACGCTTCACGAATTTG
